# Mapping scientists’ career trajectories in the survey of doctorate recipients using three statistical methods

**DOI:** 10.1038/s41598-023-34809-1

**Published:** 2023-05-19

**Authors:** Kathryn Anne Edwards, Hannah Acheson-Field, Stephanie Rennane, Melanie A. Zaber

**Affiliations:** 1 Department of Economics, Sociology, and Statistics, RAND Corporation, Santa Monica, CA USA; 2 Abt Associates, Rockville, MD USA

**Keywords:** Microbiology, Health care, Chemistry, Engineering, Physics

## Abstract

This paper investigates to what extent there is a ‘traditional’ career among individuals with a Ph.D. in a science, technology, engineering, or math (STEM) discipline. We use longitudinal data that follows the first 7–9 years of post-conferral employment among scientists who attained their degree in the U.S. between 2000 and 2008. We use three methods to identify a traditional career. The first two emphasize those most commonly observed, with two notions of commonality; the third compares the observed careers with archetypes defined by the academic pipeline. Our analysis includes the use of machine-learning methods to find patterns in careers; this paper is the first to use such methods in this setting. We find that if there is a modal, or traditional, science career, it is in non-academic employment. However, given the diversity of pathways observed, we offer the observation that traditional is a poor descriptor of science careers.

## Introduction

What is a typical career for a Ph.D. scientist? Is there a professional path that merits characterization as ‘traditional’ or ‘nontraditional?’ There are two primary ways to answer this question. The first is to have a standard path determined by preference, expectation, or a hierarchy of potential options, and the second is for a standard to be determined by patterns among observed careers. In informal parlance, this is the difference between defining traditional as the ‘ideal’ in the former or the ‘real’ in the latter. The two are neither mutually exclusive nor exhaustive, but there is an imbalance in research and discussion between the two.

Given the two potential definitions of tradition as ideal versus real, what are the ideal and real science careers? The former is not necessarily explicitly chosen or agreed upon. However, careers in science are often expressed as a pipeline: educational and workforce processes that are linear and path dependent, with attrition at every stage and each stage necessary for the next. This attrition-focused framework creates the de facto ideal career as the job representing never have leaked: a tenured research professor. The pipeline has long been criticized for its supply-side orientation, its propensity to incorrectly predict worker shortages, and its inability to account for varied career paths, among numerous other shortcomings^1–5^. Despite these criticisms, the pipeline persists as the default frame for discussing career paths in both the national STEM workforce and academia writ large, leaving a tenured academic as the ideal career. In the past few years, over a dozen new articles were published about the STEM pipeline, tenure pipeline, or the STEM-tenure pipeline^6–22^.

In this paper, we counterbalance the pipeline ‘ideal’ career with ‘real’ observations, and analyze the career trajectories of 9000 STEM Ph.D.s who graduated from a U.S. university between 2000 and 2008. We use up to nine years of longitudinal data on each STEM Ph.D. from the Survey of Doctorate Recipients (SDR) to identify, to the extent that it exists, a traditional career for a Ph.D. scientist based on observed trajectories. Our aim is to add evidence to both offset the emphasis on the pipeline and to help scientists making career choices by giving them rich information into their labor market. Aware that the ‘ideal’ career is influenced by expectations in professional culture, and that as researchers we are embedded in that culture, we apply multiple techniques in the identification of the ‘real’ career, including machine learning (ML). The use of ML methods is not completely insulated from researcher influences, but privileges algorithmic identification of data patterns.

## Data and methods

We integrate data from the Survey of Earned Doctorates (SED) and the Survey of Doctorate Recipients (SDR). The SED is a census of all graduates earning a research doctorate each year. The SED captures information about personal demographics, Ph.D. field and institution, and employment intentions (i.e., where the individual has accepted, but not started, employment). A subset of individuals from the SED are followed longitudinally in the SDR. The SDR captures information about these graduates once they enter the workforce, including type of employment and changes in employment. The SED subsample followed in the SDR is linked at the person level.

STEM fields are defined based on the NSF’s Science, Engineering and Health (SEH) definition used in the SED and SDR. This a slightly broader definition of STEM which includes the physical, life, social, engineering, and mathematical sciences as well as health sciences (e.g., physiology and nursing science), and some education and business and management degrees. Rather than construct competing or narrower definitions of STEM, we defer to the definition used by the National Science Foundation in the data but also show our results by discipline. Our sample includes doctoral recipients who responded to the SED at the time of Ph.D. conferral and in two post-conferral waves of the SDR within a timeframe specified below. These criteria capture approximately 9076 doctoral recipients in all STEM disciplines (4072 women and 5004 men) who graduated between 2000 and 2008.

There are three career observations per doctoral recipient in our data: placement (from the SED), 3–5 years after Ph.D. conferral (SDR), and 7–9 years after Ph.D. conferral (SDR). In each of the three observations, there are different employment *states*. At Ph.D. conferral, these are the placements that graduates have secured: academic, nonacademic, postdoctoral fellowship, and not working (or not placed). At 3–5 and 7–9 years from conferral, these are employment positions: tenure-track, non-tenure-track academic (research, teaching, research and teaching, neither research nor teaching), nonacademic, postdoctoral fellowship, and not working. An ordered set of employment states for an individual forms an individual *trajectory* (career path).

We use three methods to identify patterns in observed careers. First, we use an empirically driven, bottom-up categorization: we examine the most common trajectories, treating each sequence of states as a unique pattern. Second, we use a machine learning technique, algorithmic sequence analysis, which is another bottom-up approach that consolidates trajectories by shared subsequences. Finally, we classify trajectories with types defined by the pipeline to compare the applicability of the most common framework to observed careers.

To our knowledge, we are the first to apply machine-learning methods to analyzing science careers. The machine learning analysis uses the R package, TraMineR^23^. The trajectory analysis is a two-step process. First, the distance between each unique trajectory is calculated. Next, the trajectories are joined into agglomerating clusters, sequentially. To join, the algorithm iterates through each trajectory’s distance metric and joins the two closest into a cluster, eventually joining clusters with more clusters, until they form a single cluster. In agglomerative clustering, the algorithm starts with *k* = N unique trajectories, and groups the remaining trajectories iteratively by clustering the trajectories with the shortest distance between them into a group. This continues until *k* = 1 cluster remains. Agglomerative clustering seeks to minimize the distance between the trajectories within each cluster (maximizing similarity within the trajectory cluster) and maximize the distance between trajectories across clusters (maximizing the “distinctiveness” of trajectory clusters). We observe 222 unique trajectories; the first agglomeration results in 221 clusters (220 unique trajectories and a cluster of the two most similar), the next agglomeration results in 220, and then 219, and so forth, until all the trajectories are joined.

Although it is notionally a machine learning technique, the trajectory analysis is influenced by researcher decisions. The researcher must decide how to measure distance between trajectories; we use the longest common subsequence (LCS), which identifies common sequences of states among trajectories, with no preference for the trajectory having a similar start or finish. There are two alternatives: longest common prefix (LCP) and optimal matching (OM). The LCP method, as it sounds, weights the start of sequences, which we felt was inappropriate for our question. OM calculates the ‘edit distance’ between sequences based on two parameters (insert/deletion costs and substitution-cost matrix) set by the researcher; it is more fitting in longer sequences. In addition to distance metric, the researcher must decide how many clusters are appropriate in the agglomerative clustering process. We tested robustness to this choice, presenting the results from *k* = 5 clusters to easily compare with the five researcher-defined archetypes in the subsequent section. We then compare the algorithmically identified clusters of career trajectories with the other two classifications and determine how well the pipeline framework fits the evidence.

### Human subjects

The RAND Human Subjects Protection Committee (IRB) reviewed and approved this study on 6/7/2019. All methods were performed in accordance with the relevant guidelines and regulations.

The study did not collect primary data and instead used secondary data collected by the National Science Foundation National Center for Science and Engineering Statistics (NCSES). Written informed consent was provided (by NCSES) to survey respondents at each wave of participation in accordance with NSF’s IRB. All survey participants were above the age of 18 at time of survey completion.

No new or specific consent was provided for this study as it fell under approved uses of previously collected data. NSF NCSES reviewed our application for use of this data to ensure that privacy would be protected through anonymous analysis.

## Findings

### Method one: empirically defined career trajectory classifications

**Figure 1:**
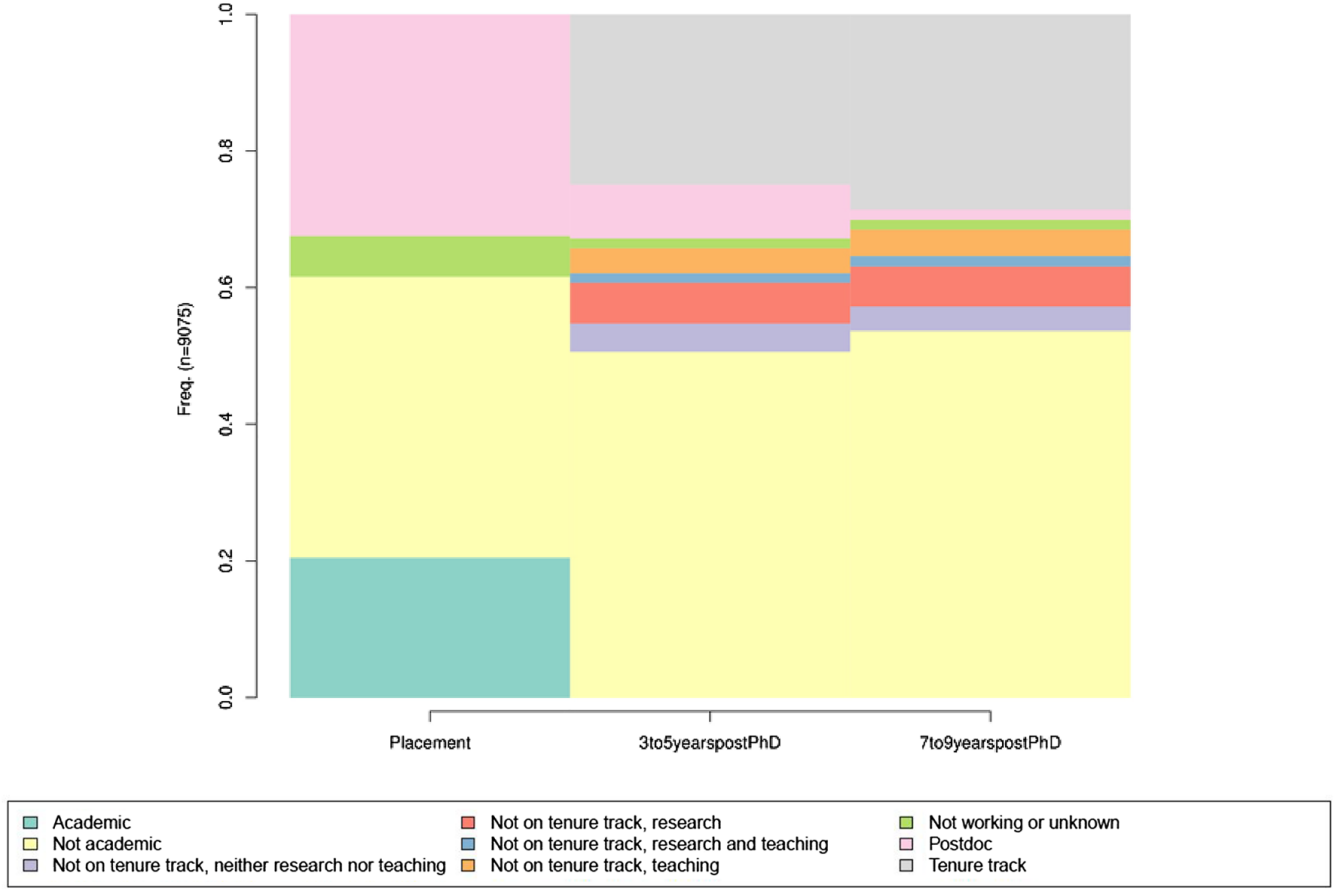
Distribution of employment of SDR sample by observation.

Figure 1 is a histogram showing the distribution of the full sample at each period by employment state. At placement, new Ph.D.s have four potential observable positions: academic, not academic, postdoc, or not working. In the years after, academic positions are divided into tenure-track or non-tenure-track, which is further divided into research positions, teaching positions, research and teaching positions, or neither research nor teaching positions. In this method, we simply observe the distribution of employment in each state and the most common paths. Non-academic is the largest group at each observation, postdocs are much less common after initial placement, and not working is relatively rare.

**Figure 2:**
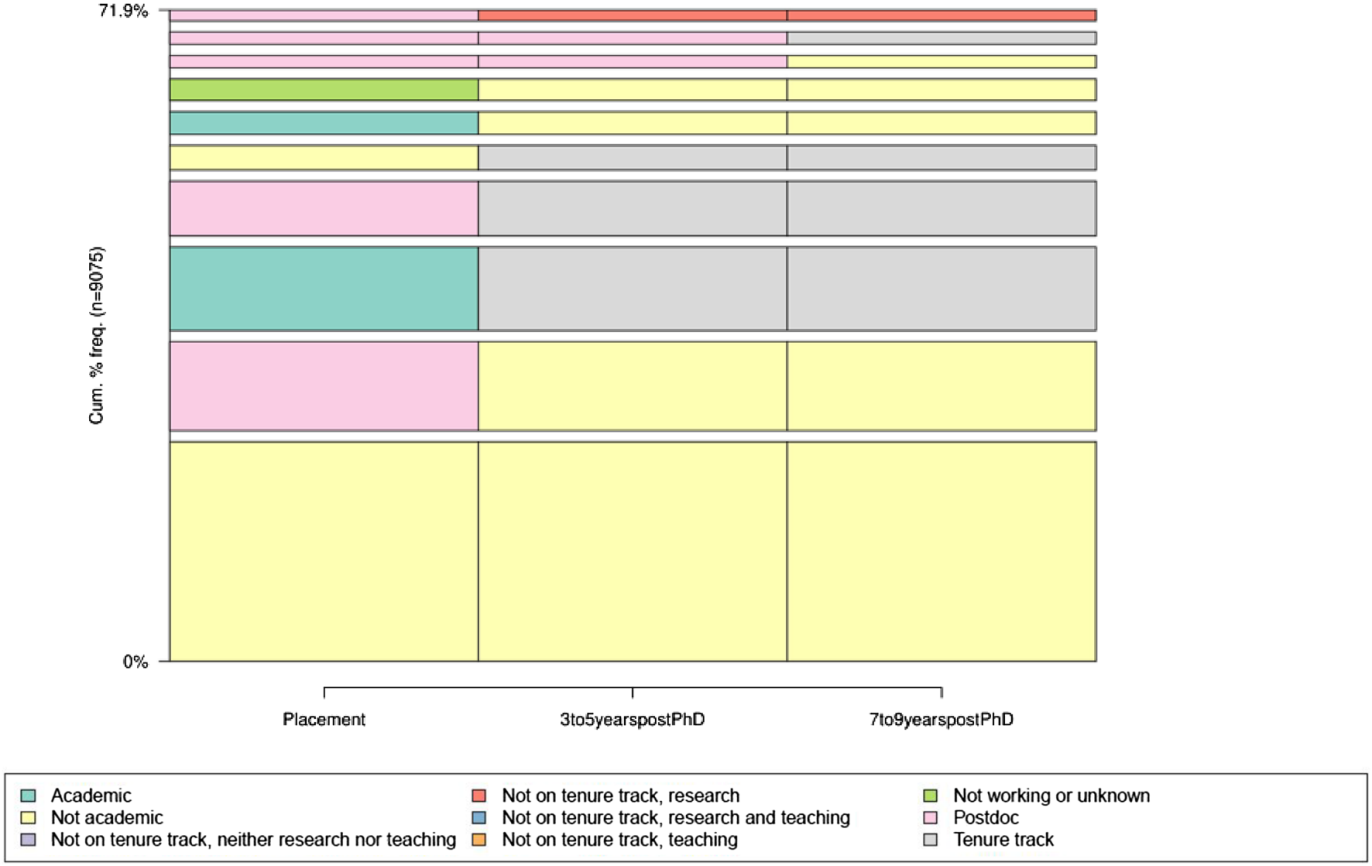
Ten most common career trajectories in the SDR.

In Fig. 2, we show the ten most common observed career trajectories (linking employment states within an individual), which account for 72% of all careers. The most common path is *Nonacademic*–*Nonacademic*–*Nonacademic*, followed by *Postdoc*–*Nonacademic*–*Nonacademic*. Again, we do not know at placement if the postdoc is academic or nonacademic. The next three are those that end up in tenure track, either because they started in an academic position, a postdoc, or a nonacademic position. These are followed more trajectories into nonacademic, one more trajectory into tenure-track, and a final trajectory into academic non-tenure-track research.

By this metric, if there is a typical career for scientists, it is to move straight from the Ph.D. to a non-academic position. However, it is indicative of the variation in pathways that the first nine most commonly observed trajectories arrive at just two outcomes—tenure-track academic and nonacademic. In addition, it is important to note that the nonacademic positions are not further delineated by whether they are research positions related to the field of PhD, and further research should explore nonacademic positions in more detail than was allowed in this project.

### Method two: algorithm-defined career trajectory classifications

**Figure 3:**
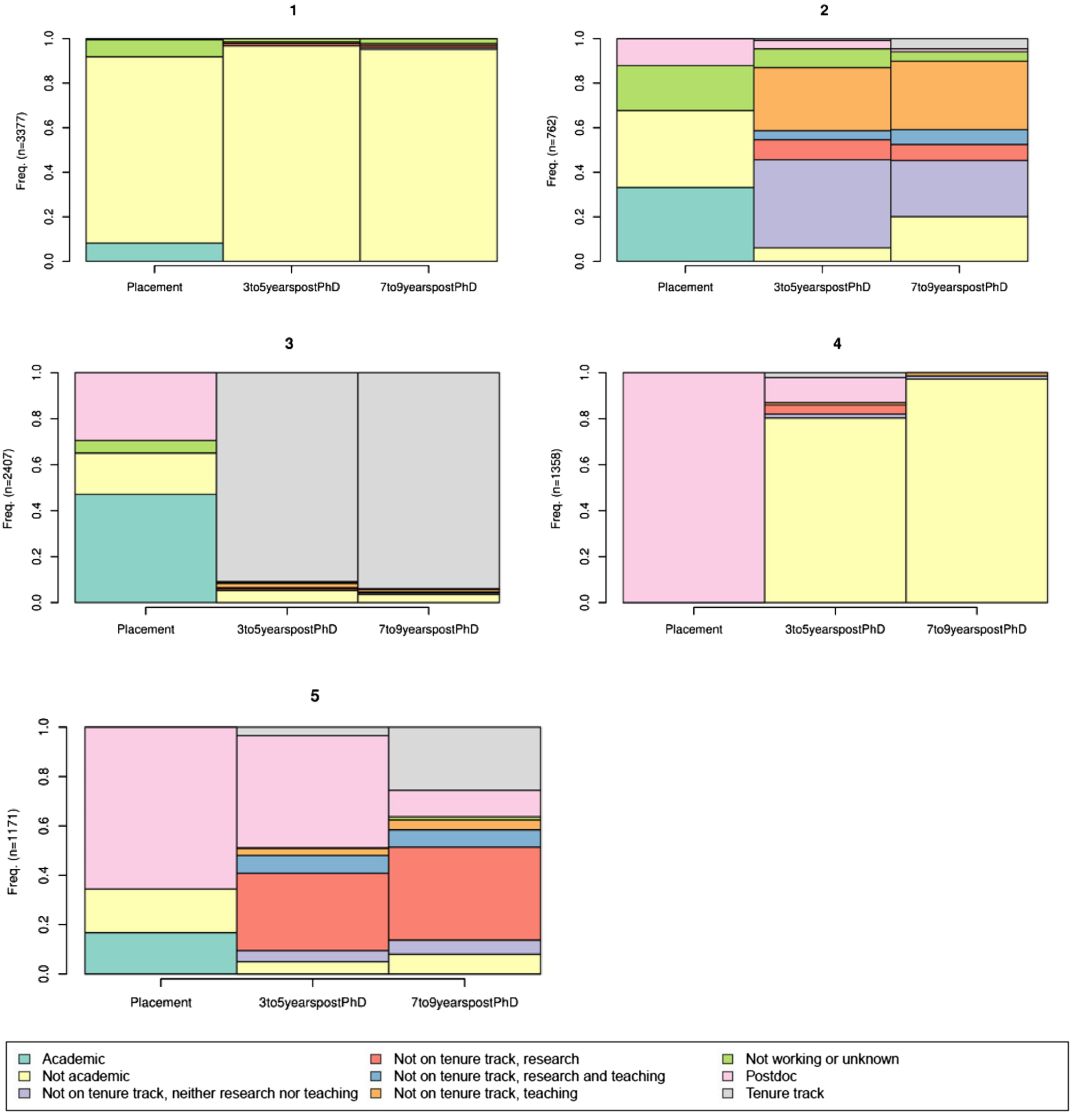
Classifications of Ph.D. career trajectories, as identified by TraMineR clustering.

Rather than counting the most common of unique pathways, in Fig. 3, we show the algorithmically derived trajectory classifications identified through the TraMineR distance and clustering sequence. Group 1, about 37% of the observed careers, is primarily not academic at each observation but includes some who are not working at placement or academic at placement. Group 2, about 8.3% of observed careers, is distinguished by non-tenure track academic employment after initial placement. Group 3, 26.5% of observed careers, is distinguished by tenure track academic employment after initial placement. Group 4, 15% of observed careers, start in a postdoc and transition to nonacademic. Group 5, 12.9% of the sample, is distinguished by multiple postdoc observations (from one or more postdoc positions) and move into academic (tenure-track or not).

Recall that the clusters group trajectories based on a distance function calculated over the total observed period. Looking to these clusters to make conclusions about typical career paths for scientists, their lack of uniformity is indicative of the diversity of career paths, even if those careers arrive at a similar place, and possibly less path dependency in trajectories.

We chose five clusters, but the algorithm creates 1–222 groups. If we were to add more clusters, each additional cluster would ‘split off’ from one of the five above, rather than rearrange the current clusters. The central result—that a bottom-up, pattern-based approach still does not yield uniformity in trajectories toward careers—is not sensitive to the choice of group number, up to a point. Having 100 clusters, for example, would result in multiple uniform paths, but the number is so high as to be uninformative.

### Method three: comparison to researcher-defined career trajectory classifications

Finally, we classify the individual career paths of Ph.D.s into four trajectories. The first three adhere to the pipeline framework: those who never enter the tenure pipeline, remaining non-academic or academic non-tenure-track for their career; those who enter the tenure pipeline but drop out; and those who are in the tenure pipeline for their career. The fourth trajectory is any remaining career paths, which by design would only include those who moved into a tenure track position from outside of a tenure track position, either a nonacademic position or a non-tenure-track academic position. For brevity, we refer to these four paths as the “Nevers”, the “Droppers”, the “Pipers”, and the “Hoppers”. In order to provide additional detail, we divide the Droppers into two groups based on when they were observed in a non-tenure-track positions—early (3–5 years) or late (7–9 years)—bringing the total trajectories to five.

The categorization requires some discretion, due to survey limitations in the SED. We cannot tell in an initial placement if a postdoctoral fellowship is with an academic or nonacademic research institution; we categorize it as part of the academic pipeline, even though some may be at a non-academic research institution. We also cannot tell in an initial academic placement if the position is tenure-track or non-tenure-track; we categorize it as part of the academic pipeline, even though some may be in non-tenure-track positions. Both of these assumptions would overestimate Droppers or Pipers and underestimate Nevers. Finally, the SED employment observation indicates where, at the time of conferral, an individual had secured a job. It is likely that graduates ‘not working’ at placement secured a job at a later date. We classify individuals who are not working as not in the academic pipeline, but individuals could have placed there after graduation.

In Table 1, we present the distribution of observed career trajectories in the SDR by the classifications defined above and the number of observations in each discipline. Less than one quarter of trajectories fit the Piper classification, adhering to the pipeline framework. Across all disciplines, 29% drop from the tenure pipeline by 7–9 years post-conferral after an initial tenure-track placement, 21% remain on the tenure track, 39% of Ph.D.s never enter the tenure pipeline, and 11% move at some point from non-tenure track to the tenure track. Combining the last two categories, 50% of observed Ph.D. careers either opt out of the academic pipeline (Nevers) or subvert it (Hoppers).

**Table 1:** Distribution of observed career trajectories in researcher-defined pipeline categories, by discipline.

	Nevers (%)	Early droppers (%)	Late droppers (%)	Pipers (%)	Hoppers (%)	Nevers + hoppers (%)
All Ph.D.s N = 9075	39	24	5	21	11	50
Agriculture *N* = 351	42	19	6	18	15	57
Biological and Biomedical Sciences *N* = 1922	24	34	11	17	13	37
Computer and Information Sciences *N* = 348	47	16	3	24	10	57
Engineering *N* = 1595	60	17	2	12	9	69
Health Sciences *N* = 599	33	21	5	25	16	49
Mathematics *N* = 347	25	17	5	39	15	40
Physical Sciences *N* = 1405	39	30	4	18	9	48
Psychology *N* = 1195	44	28	3	15	10	54
Social Sciences *N* = 1105	30	16	4	37	13	43
Other, Business, Education, Humanities *N* = 208	38	19	2	30	11	48

*Source* Authors’ analysis of the SED and the SDR.

Specific disciplines vary in this distribution. Biological and Biomedical Sciences has the highest share of Droppers at both the early (34%) and late (12%) periods. Pipers are most common in Mathematics and Social Sciences (39 and 37%), and least common in Engineering and Psychology (12 and 15%). Health sciences has the highest share of Hoppers (16%), while Engineering and Physical Sciences have the least (both 9%). Engineering and Computer and Information Sciences have the most Nevers (60 and 47%, respectively), while Biological and Biomedical Sciences have the least (24%). Given the final column of non-pipeline careers, the pipeline framework is least applicable to Engineering (69%), Agriculture (57%), and Computer and Information Sciences (57%). In only two disciplines, Mathematics and Biological and Biomedical Sciences, do more half of careers fit in the academic pipeline (63 and 60%, respectively).

The distribution looks very similar for men and women. A slightly higher percentage of women are early or late Droppers (31 and 6%, compared to 25 and 5%) but slightly lower percentage are Pipers (23% compared to 24%). Men are a higher percentage of Nevers (46 percent compared to 40%) but have identical percentage of Hoppers (13%).

In Table 2, we examine only those individuals who were in a tenure-track position 7–9 years after their Ph.D. conferral and show what percentage are Hoppers versus Pipers (these number do not match with Table 2, since Hoppers can leave a tenure track position after moving into it). Across all disciplines, 29% of all graduates are in a tenure-track position 7–9 years after Ph.D. conferral. Of those in the tenure-track, around a third, or 32%, did not follow the pipeline path, but moved at some point from a non-academic position. The discipline with the most graduates on the tenure track is Mathematics (52%), and they have a relatively low Hopper share (25%). Social Sciences is similar (49% tenure-track, 24% Hopper). Engineering, on the other hand, is only 20% tenure-track but 38% Hopper. The highest share of Hoppers in a tenure-track position is in Agriculture (41%).

**Table 2:** Share of graduates in tenure-track position 7–9 years after Ph.D. conferral, by researcher-defined pipeline categories, by discipline.

	All graduates			Tenure-track 7–9 years post		
	Total Ph.D.s	Tenure-track 7–9 years post	Share grads on tenure-track (%)	Tenure-track hoppers	Tenure-track pipers	Share hoppers in tenure-track (%)
All Ph.D.s	9075	2647	29	841	1806	32
Agriculture	351	107	30	44	63	41
Biological and Biomedical Sciences	1922	518	27	187	331	36
Computer and Information Sciences	348	117	34	32	85	27
Engineering	1595	315	20	119	196	38
Health Sciences	599	235	39	84	151	36
Mathematics	347	179	52	44	135	25
Physical Sciences	1405	359	26	104	255	29
Psychology	1195	276	23	96	180	35
Social Sciences	1105	541	49	131	410	24
Other, Business, Education, Humanities	208	83	40	20	63	24

*Source* Authors’ analysis of the SDR.

The differences in the share of tenure-track who are Hoppers does not vary by gender when considering all disciplines (both are 32%). In certain disciplines, a higher share of tenure-track men are Hoppers (Agriculture, Biological and Biomedical Sciences, Engineering, Physical Sciences, Social Sciences), while in others tenure-track women have a higher share of Hoppers (Computer and Information Sciences, Health Sciences, Mathematics, Psychology, and Other). To explore whether there was a pattern within the Hoppers subgroup, we regressed a binary variable indicating whether an individual was a Hopper on: individual descriptors (gender, age at conferral, marital status at conferral), Ph.D. institution descriptors (public, HBCU), Ph.D. department descriptors (the average number of publications per faculty member, number of programs, average number of citations per publication, percent of faculty that are female), and economic descriptors (an indicator for graduating during a recession). We did this for the full population, and the 7–9 year tenure track population, and for separate disciplines. Most predictors were not significant in most specifications, and few were significant across specification. We do not show the regression output, both for brevity and because we do not want to risk overinterpreting weak results.

### Synthesis of findings: real and ideal

Each method in our analysis was chosen deliberately. Method 1, which is akin to an accounting, establishes modal career trajectories and that the academic “pipeline” is not included in them. Method 2, the ML trajectory analysis, offers a way to approach the data while minimizing the influence of our own experiences or expectations. It does not require path dependence, though it could detect it, and does not rank outcomes, only differentiates between them as separate states. In effect, this removes what “should” happen in scientist careers and what is “best” from the analysis of the data; it is simply an analysis of sequential states. Method 3 offers a contrast to 2, where “should” and “best” are applied to careers via the pipeline categorization, allowing for a clear identification of its failures as a heuristic.

Our analysis of observed careers finds that there is no single traditional scientist career; rather, trajectories evince numerous pathways. The exception is those that spend their careers outside of academia (from method 1) have little pathway variation. However, our grouping of ‘all non-academic’ is blunt; with data that supported more non-academic categories, we suspect we would find similar diversity in pathways. In addition, we also find (from method 2), when the characterization was on commonality between paths, rather than path-dependence, that there are diverse ways to arriving at similar positions. Even within tenure-track academics 7–9 years after PhD conferral (from method 3), one-third had not followed the pipeline path.

Our findings suggest that the prevailing notion of an ideal career in science as an academic who followed the tenure-track—the “pipeline”—has two shortcomings: it is not a common career, and it excludes alternative pathways to tenure. The pipeline is not readily applicable to scientist careers, and when it is applied, it renders many potentially satisfying careers as suboptimal (to the extent “leaks” are seen as negative outcomes).

We are not the first to find that the pipeline has shortcomings. The pipeline is a supply-focused concept; in order for there to be sufficient workers at the end, there must be a large enough initial stock. Its specific application to science, technology, engineering, and math (STEM) workers originated in the 1970s, and, beginning in the 1980s, was used by the National Science Foundation to predict potential future worker shortages in professions key to national competitiveness^2^. As early as 1992, the House Committee on Science, Space, and Technology held hearings on NSF-produced pipeline studies and noted that criticism of the model was disregarded^1,24^. Other researchers have noted that the prediction for scientist shortages are not accurate^25^, partly because a separate criticism, that the pipeline does not take into account varied career paths^2,26^.

The pipeline’s emphasis on supply-side factors often makes it a default explanation for shortages, giving it a central role in discussions of the gender or racial composition of academic departments^26^. The lack of diversity at senior, tenured levels is often attributed to how slowly changes make their way through the pipeline, and the relative “leak” rates of different groups are used to identify potential interventions. Again, the appropriateness of the pipeline has also long been criticized in this context as well, for ignoring other structural and cultural barriers to success, enabling inaction, and even serving as a deterrent of specific career paths^4,5^.

Our findings of numerous pathways not only add to the pipeline criticism, but also suggest an alternative metaphor: a lattice, akin to a netting. A lattice supports lateral and forward movement, removes the notion of ‘leaking,’ and emphasizes *numerous* pathways over *preferred* pathways. Lattice pathways, while not required to be unidirectional, can still include barriers to progress or participation but offer more than one means of identifying them. Further, by eliminating path dependence, the lattice also subverts the debate of predictive shortages, as each pathway creates a potential pool for supply. It also naturally enumerates policy interventions. A pipeline’s solution centers around more supply in a single path; a lattice identifies numerous supplying paths, or channels, that can then be improved or augmented. As a career framework, the lattice could also provide context for future studies of career pathways that investigate trajectories as they vary by gender, race, immigration status, Ph.D. institution, or other relevant demographic and academic characteristics, or how trajectories are influenced by constraints and preferences, such as family-partner coordination or geographic preferences.

## Conclusion

This paper examines the career trajectories of Ph.D. scientists for up to 9 years after their Ph.D. conferral in an effort to understand what is a traditional career for scientists. Based on commonality alone, the most traditional career is to work outside of academia upon graduation. However, further examination and classification of career trajectories instead emphasizes the diversity of career pathways into employment states. We found this was the case even for tenure-track academics, a third of whom did not follow the pipeline process. This raises the question of whether the notion of ‘traditional’ is well applied to scientists.

Our findings have limitations. First, we do not observe full careers; we focus on the first 7–9 years after Ph.D. conferral. Second, our understanding of career pathways does not permit a detailed investigation of pathways with non-academic employers. In particular, we cannot discern job- or employer-specific tenure, whether the non-academic employment is in research or related to the individual’s Ph.D. training, nor if non-academic employment is in fact entrepreneurship. Third, our sample, while large, did not have sufficient power to detect demographic or academic differences in regression predictions of trajectory type.

The findings of this paper motivate additional research into discipline-specific analysis of trajectories and how they relate to labor markets, such as the number of tenure-track positions relative to Ph.D. students, the source of funding for graduate students and faculty, and the pay and benefits of non-academic positions. In addition, the findings motivate a mapping of non-academic employment with more detailed job information.

## Data Availability

The analyses in this study were conducted using restricted-use data files from the Survey of Earned Doctorates and the Survey of Doctoral Recipients under data license #156. Data from these sources cannot be shared publicly because of the restricted use guidelines put in place by NCSES. IPEDS data is publicly available and accessible at https://nces.ed.gov/ipeds/use-the-data, and data from the National Research Council is available at https://www.nap.edu/download/12994. Our data are restricted-use and we cannot provide even a minimal data set due to the sensitivity of the data and the terms of our usage license. A project application for restricted access to NCSES data (such as the SED/DRF and SDR used in this project) can be submitted through this NSF portal. The application needs to include the data requirements (data products, years), a research plan, and a list of the specific restricted-use variables needed (here's a recent data dictionary). After the application is approved, the researcher must complete a notarized license application, which includes NCSES security training. Upon license approval, researchers are able to access restricted-use data, either by remoting into a secure portal or visiting a Federal Statistical Research Data Center. The application and licensing process is currently overseen by Darius Singpurwalla, who can be reached at NCSES_Licensing@nsf.gov or dsingpur@nsf.gov.
